# Bearing Fault Diagnosis Using Refined Composite Generalized Multiscale Dispersion Entropy-Based Skewness and Variance and Multiclass FCM-ANFIS

**DOI:** 10.3390/e23111510

**Published:** 2021-11-14

**Authors:** Mostafa Rostaghi, Mohammad Mahdi Khatibi, Mohammad Reza Ashory, Hamed Azami

**Affiliations:** 1 Modal Analysis (MA) Research Laboratory, Faculty of Mechanical Engineering, Semnan University, Semnan 35131-19111, Iran; rostaghi@semnan.ac.ir (M.R.); mashoori@semnan.ac.ir (M.R.A.); 2 Department of Neurology and Massachusetts General Hospital, Harvard Medical School, Charlestown, MA 02129, USA; hmdazami@gmail.com

**Keywords:** dispersion entropy, fault diagnosis, refined composite generalized multiscale dispersion entropy (RCGMDispEn), bearing, multiclass FCM-ANFIS

## Abstract

Bearing vibration signals typically have nonlinear components due to their interaction and coupling effects, friction, damping, and nonlinear stiffness. Bearing faults affect the signal complexity at various scales. Hence, measuring signal complexity at different scales is helpful to diagnosis of bearing faults. Numerous studies have investigated multiscale algorithms; nevertheless, multiscale algorithms using the first moment lose important complexity data. Accordingly, generalized multiscale algorithms have been recently introduced. The present research examined the use of refined composite generalized multiscale dispersion entropy (RCGMDispEn) based on the second moment (variance) and third moment (skewness) along with refined composite multiscale dispersion entropy (RCMDispEn) in bearing fault diagnosis. Moreover, multiclass FCM-ANFIS, which is a combination of adaptive network-based fuzzy inference systems (ANFIS), was developed to improve the efficiency of rotating machinery fault classification. According to the results, it is recommended that generalized multiscale algorithms based on variance and skewness be examined for diagnosis, along with multiscale algorithms, and be used to achieve an improvement in the results. The simultaneous usage of the multiscale algorithm and generalized multiscale algorithms improved the results in all three real datasets used in this study.

## 1. Introduction

Bearings are among the most important and useful rotating machinery components [1]. A lack of timely diagnosis and replacement of bearings can disrupt the functionality of machinery. For instance, 40–50% of all electrical motor failures are associated with bearing failure [2]. Prompt fault detection in bearings can reduce financial loss and health risks.

The vibration signals of bearings usually exhibit a nonlinear behavior due to the effects of coupling and nonlinear interactions, friction, damping, and stiffness [3], and faults at different signal scales impact signal complexity. Hence, measurements of signal complexity at various scales can contribute to diagnosis and, thus, are commonly used.

Entropy is a measure of the disorder and predictability of the signal. It is one of the most powerful concepts used to evaluate signal characteristics [4]. Several entropies have been introduced to date, such as sample entropy (SampEn) and permutation entropy (PerEn). We recently introduced dispersion entropy (DispEn) [5] and demonstrated its advantage over PerEn and SampEn [5]. In addition to being fast, DispEn can provide a better representation of dynamic signal changes. PerEn considers only the order of the amplitudes with respect to each other, but DispEn takes into account the values of the amplitudes. Unlike SampEn, DispEn is also defined in short series [6]. Moreover, DispEn is relatively insensitive to noise [3]. Rostaghi et al. investigated the potential applications of DispEn in rotating machinery diagnosis and demonstrated its superiority over PerEn and approximate entropy (ApEn) [3]. Liu et al. combined DispEn and wavelet packets to extract the features used for bearing diagnosis [7]. They calculated the DispEn of each wavelet packet. Li et al. computed the intrinsic mode function (IMF) components of the signals via an improved complete ensemble empirical mode decomposition and used DispEn of the first few IMF components for bearing diagnosis [8]. Zhenzhen et al. employed variational mode decomposition (VMD) and DispEn for bearing diagnosis [9].

Disorder and complexity have different physical meanings [10,11]. Accordingly, conventional entropies cannot represent complexity without using other algorithms. Therefore, Costa et al. introduced the multiscale algorithm in 2002 to show complexity and analyze non-stationary and nonlinear signals [12]. They utilized this algorithm for SampEn. Subsequently, this algorithm was used for various entropies and enhanced multiple times. Aziz et al. introduced multiscale permutation entropy (MPerEn) [13]. Wu et al. introduced refined composite multiscale entropy (RCMSampEn) [14], Humeau-Heurtier et al. refined composite multiscale permutation entropy (RCMPerEn) [15], and Azami et al. refined composite multiscale dispersion entropy (RCMDispEn) [16].

Wang et al. utilized MDE for feature extraction in bearing diagnosis [17]. Congzhi et al. calculated the RCMDispEn of vibration signals and classified them using the support vector machine (SVM) for bearing diagnosis [18]. Zhang et al. utilized RCMDispEn and an improved SVM based on the whale optimization algorithm for fault detection of rolling bearings [19]. Lou et al. employed the RCMDispEn and the deep belief network-extreme learning machine optimized by the improved firework algorithm for rolling bearing sub-health recognition [20].

Various techniques have been used along with RCMDispEn for bearing diagnosis. These techniques include the fast ensemble empirical mode decomposition [21], adaptive sparest narrow-band decomposition [22], improved empirical wavelet transform [23,24], VMD [25], and improved VMD (IVMD) [26]. 

Costa et al. introduced generalized multiscale entropy (GMSE) in 2015 [27]. Generalized algorithms use other statistical properties, such as variance, for coarse-graining. Costa et al. specifically proposed and utilized the standard deviation (SD) and variance [27]. Wei et al. stated that, unlike the first moment, the second moment simultaneously separates the high and low frequency contents during coarse-graining [28], and employed variance-based generalized multiscale fuzzy entropy for diagnosis in rotating machinery [28]. Zheng et al. utilized generalized composite multiscale permutation entropy-based variance and the Laplacian score for bearing diagnosis [29]. Liu et al. detected bearing faults using generalized composite multiscale amplitude-aware permutation entropy-based variance and dual-tree complex wavelet packet transform [30]. 

Because of the advantages of DispEn-based algorithms over the SampEn-, FuzEn-, and PerEn-based algorithms [6], the present paper investigates refined composite generalized multiscale dispersion entropy (RCGMDispEn) based on variance and skewness with RCMDispEn for bearing fault diagnosis. It is worth mentioning that generalized multiscale dispersion entropy is proposed in this study for the first time to probe the properties of time series related to higher moments (the second and third moments, i.e., variance and skewness).

A combination of several classifiers was used to overcome the limitations of each classifier and achieve higher efficiency [31,32,33]. In numerous studies, several classifiers have been used with a classifier utilizing the results of the other classifiers for final classification [32,34,35]. Belaout et al. combined several Sugeno ANFIS to construct an output vector and introduced a multiclass ANFIS based on the winner-takes-all rule [36]. Similarly, multiclass FCM-ANFIS was used in this study to classify different kinds of faults.

The rest of the paper is organized as follows. Section 2.1 reviews the theory of DispEn, and Section 2.2 and Section 2.3 introduce the calculation of GMDispEn and RCGMDispEn, respectively. Section 3 introduces the theory behind combining ANFIS networks. In Section 4, RGMDispEn and GMDispEn methods are compared to MDispEn and RCMDispEn, respectively, in terms of diagnosis capability in simulated bearing signals. Section 5 uses three different datasets to demonstrate that simultaneously using RCGMDispEn and RCMDispEn in practical applications can provide better efficiency than MDispEn and RCMDispEn. Finally, Section 6 concludes the paper.

## 2. Generalized Refined Composite Multi-Scale Dispersion Entropy

### 2.1. Dispersion Entropy

The DispEn for the time series $x=\text{\{}x_{1},\;x_{2},\;x_{3},\ldots ,x_{N}\text{\}}$ with a length of *N* can be calculated in six steps [5]:

Step 1. The signal is normalized between 0 and 1. The series $y=\{y_{1},y_{2},\ldots ,y_{N}\}$ is obtained according to (1) from the normal cumulative distribution function (NCDF) of the series x:

(1) $$y_{i}=\frac{1}{\sigma \sqrt{2\pi }}\int_{-\infty }^{x_{i}}e^{\frac{-{\left(t-\gamma \right)}^{2}}{2\sigma^{2}}}dt$$

Here, σ and γ denote the SD and mean value of the time series x, respectively. 

Step 2. Each member of the time series **y** is mapped to an integer between 1 and *c* (Equation (2)):

(2) $$z_{i}^{c}=\mathrm{round}(c.y_{i}+0.5)$$

*c* is the class parameter and indicates the number of classes that can be members of the time series $z^{c}$. $z_{i}^{c}$ is the *i*^th^ member of the classified series $z^{c}$.

Step 3. All the template vectors $z_{j}^{m,c}$ (j=1,2,…,N−(m−1)d) are created as follows:

(3) $$z_{j}^{m,c}=\{z_{j}^{c},z_{j+d}^{c},\ldots ,z_{j+(m-1)d}^{c}\}$$

where *m* and *d*, respectively, denote the embedding dimension and time delay. The embedding dimension is the dimension of the state space used for reconstruction.

Step 4. Each series $z_{j}^{m,c}$ is mapped to a pattern $\pi_{v_{0}v_{1}\ldots v_{m-1}}$ based on its values, while the following holds:

(4) $$z_{j}^{c}=v_{0},z_{j+(1)d}^{c}=v_{1},z_{j+(2)d}^{c}=v_{2},\ldots ,z_{j+(m-1)d}^{c}=v_{m-1}$$

The number of possible dispersion patterns that can be attributed to each series $z_{j}^{m,c}$ is equal to $c^{m}$, because each $z_{j}^{m,c}$ has *m* members, and each of them can be an integer from 1 to *c* [5]. 

Step 5. For every $c^{m}$ dispersion patterns $\pi_{v_{0}v_{1}\ldots v_{m-1}}$, the relative frequency is obtained using Equation (5); i.e., the number of dispersion patterns $\pi_{v_{0}v_{1}\ldots v_{m-1}}$ that are attributed to the series $z_{j}^{m,c}$ is divided by the total number of *m*-dimensional series created.

(5) $$p(\pi_{v_{0}v_{1}\ldots v_{m-1}})=\frac{Number\{t|t\leq N-(m-1)d,z_{j}^{m,c}\mathrm{has}\;\mathrm{type}\;\pi_{v_{0}v_{1}\ldots v_{m-1}}\}}{N-(m-1)d}$$

$p(\pi_{v_{0}v_{1}\ldots v_{m-1}})$ is the probability of dispersion pattern $\pi_{v_{0}v_{1}\ldots v_{m-1}}$.

Step 6. DispEn with the embedding dimension *m* and number of classes *c* is calculated according to Equation (6):

(6) $$DispEn(x,m,c,d)=-\sum p(\pi_{v_{0}v_{1}\ldots v_{m-1}})\ln p(\pi_{v_{0}v_{1}\ldots v_{m-1}})$$

To calculate the normalized DispEn (NDispEn) according to Equation (7), DispEn is divided by the largest possible DispEn.

(7) $$NDispEn(x,m,c,d)=\frac{DispEn(x,m,c,d)}{\ln(c^{m})}$$

When *m* or *c* is too large, the computation time is high, although it makes the DispEn values more reliable [5]. In addition, if the embedding dimension *m* is too small, the dynamic changes may not be detected in the signal, whereas large *m* may cause DispEn to be unable to observe small variations [5]. Based on the abovementioned facts and previous studies [3,5], the parameters *m* = 2 and *c* = 8 were used to calculate DispEn. 

### 2.2. Generalized Multiscale Dispersion Entropy

Multiscale dispersion entropy (MDispEn) and generalized MDispEn (GMDispEn) compute DispEn in several consecutive scales based on the first and other momenta. The *n*^th^-moment-based generalized MDispEns are displayed as GMDispEn_n_. They are implemented as follows:

The signal is coarse-grained up to where the time series $y^{n,(\tau )}$, which is the time series **x** with the scale τ and the *n*^th^ moment, is constructed [19]:

For MDispEn, based on the first moment:

(8) $$y_{j}^{1,(\tau )}=\frac{1}{\tau }\sum_{i=(j-1)\tau +1}^{j\tau }x_{i},\;1\leq j\leq \frac{N}{\tau }$$

For GMDispEn_2_, based on the second moment (variance):

(9) $$y_{j}^{2,(\tau )}=\frac{1}{\tau }\sum_{i=(j-1)\tau +1}^{j\tau }\;(x_{i}-{\bar{x}}_{j}{)}^{2},\;1\leq j\leq \frac{N}{\tau }$$

For GMDispEn_3_, based on the third moment (skewness):

(10) $$y_{j}^{3,(\tau )}=\frac{1}{\tau }\sum_{i=(j-1)\tau +1}^{j\tau }\;(x_{i}-{\bar{x}}_{j}{)}^{3},\;1\leq j\leq \frac{N}{\tau }$$

where ${\bar{x}}_{j}=\frac{1}{\tau }\sum_{i=(j-1)\tau +1}^{j\tau }x_{i}$.

The DispEn of the signal $y^{n,(\tau )}$ is computed. Here, the mean and the SD of the main signal are used for mapping based on the NCDF before coarse-graining. This approach is similar to keeping *r* constant while calculating the multiscale entropy (MSE) such that *r* = 0.15 × D (original signal) for all scales.

With a change in τ, often carried out by adding 1 to τ, Steps 1 and 2 are repeated until the desired scale is reached.

For DispEn, the parameters must be set in such a way that the number of possible dispersion patterns becomes smaller than the signal length $\left(c^{m}<L\right)$. Because the signal length for GMDispEn is reduced to $\left\lfloor \frac{L}{\tau_{\max}}\right\rfloor$ due to coarse-graining, $c^{m}<\left\lfloor \frac{L}{\tau_{\max}}\right\rfloor$ is recommended for GMDispEn.

### 2.3. Generalized Refined Composite Multi-Scale Dispersion Entropy

In the calculation of the RCMDispEn and the *n*^th^-moment-based RCGMDispEn (RCGMDispEn_n_) with a scale factor of τ, τ different time series are constructed by coarse-graining based on the first and higher momenta in order and with different starting points. The relative frequency of the dispersion patterns is calculated from every τ time series. The *k*^th^ coarse-grained time series $x_{k}^{n,(\tau )}=\left\{x_{k,1}^{n,(\tau )},x_{k,2}^{n,(\tau )},\ldots \right\}$ from the series x is obtained based on the *n*^th^ moment and the scale τ as follows:

(11) $$x_{k,j}^{1,(\tau )}=\frac{1}{\tau }\sum_{i=k+\tau (j-1)}^{k+\tau j-1}x_{i},1\leq j\leq \frac{N}{\tau },\;1\leq k\leq \tau$$

(12) $$x_{k,j}^{2,(\tau )}=\frac{1}{\tau }\sum_{i=k+\tau (j-1)}^{k+\tau j-1}(x_{i}-{\bar{x}}_{k,j}{)}^{2},1\leq j\leq \frac{N}{\tau },\;1\leq k\leq \tau$$

(13) $$x_{k,j}^{3,(\tau )}=\frac{1}{\tau }\sum_{i=k+\tau (j-1)}^{k+\tau j-1}(x_{i}-{\bar{x}}_{k,j}{)}^{3},1\leq j\leq \frac{N}{\tau },\;1\leq k\leq \tau$$

where ${\bar{x}}_{k,j}=\frac{1}{\tau }\sum_{i=(j-1)\tau +k}^{j\tau +k-1}x_{i}$.

Hence, for every scale factor, RCGMDispEn_n_ is defined as follows:

(14) $$RCGMDispEn_{n}(x,m,c,d,\tau )=-\sum \bar{p}(\pi_{v_{0}v_{1}....v_{m-1}}).\ln(\bar{p}(\pi_{v_{0}v_{1}....v_{m-1}}))$$

where $\bar{p}(\pi_{v_{0}v_{1}....v_{m-1}})=\frac{1}{\tau }\sum_{k=1}^{\tau }p_{k}^{\left(\tau \right)}(\pi_{v_{0}v_{1}....v_{m-1}})$. $p_{k}^{\left(\tau \right)}(\pi_{v_{0}v_{1}....v_{m-1}})$ is the relative frequency of the dispersion pattern $\pi_{v_{0}v_{1}....v_{m-1}}$ in the time series $x_{k}^{n,(\tau )}$.

In RCGMDispEn, τ coarse-grained time series with a length of $\left\lfloor \frac{L}{\tau_{\max}}\right\rfloor$ are considered. Thus, the total number of samples calculated in RCGMDispEn is $\tau \times \left\lfloor \frac{L}{\tau_{\max}}\right\rfloor \approx L$. Therefore, RCGMDispEn with a length of $c^{m}<L$ produces reliable results. This special property is significant in short-length signals.

It must be noted that the scale starts from 2 for calculating GMDispE_2_ and RCGMDispE_2_, and from 3 for calculating GMDispE_3_ and RCGMDispE_3_ [37,38].

## 3. Multiclass Adaptive Neuro-Fuzzy Classifier

### 3.1. Adaptive Neuro-Fuzzy Inference System (ANFIS)

ANFIS is a fuzzy model expressed in the form of a neural network [39]. It is characterized by a synergic collaboration between the fuzzy theory and neural networks. ANFIS combines a treatment of the uncertainty and interpretability of fuzzy systems with the learning capability of neural networks [40,41]. It utilizes neural network learning algorithms to estimate the parameters of the fuzzy model [42].

An ANFIS structure is composed of five layers with nodes in the same layer possessing the same function family, as explained below:

First layer: In this layer, the membership degrees of each input with respect to the membership functions are calculated. Various membership functions can be employed here. Because the partial derivatives of the Gaussian function parameters are smooth, the Gaussian function was used in this research:

(15) $$\mu_{ij}(x_{sj})=\exp(-\frac{{\left(x_{sj}-c_{ij}\right)}^{2}}{2\sigma_{ij}^{2}})$$

Here, $\mu_{ij}$ represents the membership of the Gaussian function with respect to the *i*^th^ rule and *j*^th^ feature, and $x_{sj}$ denotes the *j*^th^ feature of the *s*^th^ sample. The parameters $c_{ij}$ and $\sigma_{ij}$ respectively represent the center and width of the Gaussian function.

Second layer: In this layer, the fuzzy implication in each node is computed using the input membership degrees. $\theta_{ic}$, which is the implication of the *i*^th^ rule for the sample $x_{c}$, is obtained as follows:

(16) $$\theta_{ic}=\prod_{j=1}^{N}\mu_{ij}(x_{cj})$$

Here, *N* denotes the number of input features $x_{c}$.

Third layer: In this layer, the ratio of the implication of the rule associated with every node to the total rule implications is computed in every node. The normalized implication of the *i*^th^ node ($\bar{\theta_{ic}}$) is determined as follows:

(17) $$\bar{\theta_{ic}}=\frac{\theta_{ic}}{\sum_{i=1}^{M}\theta_{ic}}$$

*M* equals the number of rules.

Fourth layer: The nodes in this layer are adaptive nodes. The weighted output of each node in this layer $\varphi_{ic}$ is obtained by multiplying $\bar{\theta_{ic}}$ with a corresponding first-degree polynomial ($f_{ic}$):

(18) $$\varphi_{ic}=\bar{\theta_{ic}.}f_{ic}$$

The coefficients of the polynomial $f_{ic}$ and the coefficients $c_{ij}$ and $\sigma_{ij}$ corresponding to the first layer are updated by the learning algorithms of the neural network.

Fifth layer: This layer contains a single fixed node that calculates the output $f_{out}$:

(19) $$f_{out}(x_{c})=\sum_{i=1}^{M}\varphi_{ic}=\sum_{i=1}^{M}\bar{\theta_{ic}}.f_{ic}$$

In this paper, fewer fuzzy rules were obtained by using fuzzy c-means (FCM), which automatically constructs a fuzzy rule base for ANFIS. A combination of the least-squares method and the backpropagation gradient descent method was used to adjust the membership functions and other parameters.

### 3.2. Fuzzy C-Means

FCM is a clustering algorithm that assigns each data point to a cluster with a specific degree of membership. Dunn introduced this algorithm [43] and Bezdek subsequently improved it [44,45].

FCM employs the minimization of the objective function [45]:

(20) $$J_{m}(u,c)={\sum_{j=1}^{D}\sum_{i=1}^{N}\mu_{ij}^{m}\left\|x_{i}-c_{j}\right\|}^{2}$$

where *N* and *D* represent the number of data points and clusters, respectively. Moreover, $x_{i}$ denotes the *i*^th^ data point, and $c_{j}$ is the center of the *j*^th^ cluster. $\mu_{ij}$ is the membership degree of $x_{i}$ with respect to the *j*^th^ cluster, and m represents the fuzziness parameter. ‖.‖ denotes the Euclidean distance.

The objective function is minimized via an iterative process of updating the fuzzy membership degrees and the cluster centers [46]. The steps to implementing FCM are as follows [45,47]:

1-The membership degrees of the clusters $\mu_{ij}$ are randomly initialized.

2-The centers and membership degrees of the clusters are calculated as below:

(21) $$c_{j}=\frac{\sum_{i=1}^{D}\mu_{ij}^{m}x_{i}}{\sum_{i=1}^{D}\mu_{ij}^{m}}$$

3-The membership degrees of the clusters are updated as follows:

(22) $$\mu_{ij}=\frac{1}{\sum_{k=1}^{N}{(\frac{\left\|x_{i}-c_{j}\right\|}{\left\|x_{i}-c_{k}\right\|})}^{\frac{2}{m-1}}}$$

4-The objective function $J_{m}$ is computed.

5-Steps 2 to 4 are repeated until a minimum threshold for the objective function or the maximum number of iterations is reached.

### 3.3. Multiclass FCM-ANFIS 

A combination of several classifiers is used to overcome the limitations of each classifier and achieve higher efficiency [34]. Each Sugeno ANFIS may be considered a binary classifier, and a set of them can be used in multiclass classification problems [36]. The final inference can take place via the winner-takes-all rule [36]. Accordingly, multiclass FCM-ANFIS was employed in this research. In this technique, every FCM-ANFIS examines the possibility of assigning a specific class to each input sample. Specifically, the *k*^th^ FCM-ANFIS examined the possibility of assigning the class *k* to the inputs, and the target was considered to be 1 for the class *k* and zero for the rest of the classes. The final class is the one whose FCM-ANFIS has the largest output:

$$\max([output({\mathrm{FCM-ANFIS}}_{1}),\ldots ,output({\mathrm{FCM-ANFIS}}_{N})])=output({\mathrm{FCM-ANFIS}}_{k})\Rightarrow \mathrm{Final}\;\mathrm{output}=\mathrm{Class}\;k$$

**Figure 1:**
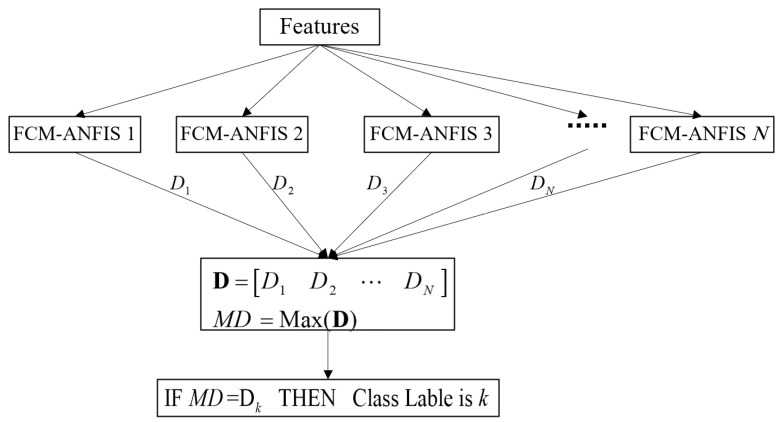
Implementation of multiclass FCM-ANFIS.

Figure 1 displays the implementation of multiclass FCM-ANFIS.

## 4. Analysis of a Simulated Bearing Signal

The vibration signal of ball bearing with an outer race fault was simulated as follows:

(23) $$x(t)=x_{\mathrm{series}\;\mathrm{of}\;\mathrm{impulses}}+x_{\mathrm{harmonic}\;\mathrm{component}}+n(t)$$

where $x_{\mathrm{series}\;\mathrm{of}\;\mathrm{impulses}}$ and $x_{\mathrm{harmonic}\;\mathrm{component}}$ represent the impulse series and the harmonic series, respectively, and *n*(*t*) denotes the noise. 

Based on previous studies [48,49], $x_{\mathrm{series}\;\mathrm{of}\;\mathrm{impulses}}$ was modeled using Equation (24):

(24) $$x_{\mathrm{series}\;\mathrm{of}\;\mathrm{impulses}}=\sum_{k=1}^{m}\sum_{n=1}^{n^{\prime }}Ae^{-2\xi \pi f_{n}(t-\frac{k}{f_{f}}-\sum_{i=1}^{k}\tau_{k})}\sin(2\pi f_{n}\sqrt{1-\xi^{2}}(t-\frac{k}{f_{f}}-\sum_{i=1}^{k}\tau_{k}))$$

$f_{n}$ represents the resonance frequencies of the bearing, which are significantly higher than the fault frequency $f_{f}$. $\tau_{k}$ represents a small random change in the interval between two impulses. The ball slipping effect changes the period randomly to $\frac{k}{f_{f}}-\tau_{k}$. Hence, for every *k*, $\tau_{k}$ was considered to be a random number from a normal distribution with a zero mean and standard deviation of $\sigma_{\tau }=0.005\times \frac{1}{f_{f}}$.

Two sinusoidal functions were employed for the harmonic part of Equation (23) [50,51]:

(25) $$x_{\mathrm{harmonic}\;\mathrm{component}}=\sum_{m=1}^{2}B_{m}\sin(2\pi mf_{r}t)$$

In this simulation, the characteristic frequency of the fault and the damping factor were assumed to be $f_{f}=100\;\mathrm{Hz}$ and ξ=0.03. Moreover, $f_{1}=2\;\mathrm{KHz},f_{2}=3.5\;\mathrm{KHz}$ represent the resonance frequencies of the bearing, and A=1 denotes the magnitude of the impulse amplitude, which is a measure of the damage intensity. In addition, the rotor frequency was considered to be $f_{r}=30\;\mathrm{Hz}$, and $B_{1}=0.2$ and $B_{2}=0.12$ represent the amplitudes of the first and second harmonics of the rotor, respectively. The signal of a healthy bearing was modeled by eliminating the fault impulses. 

**Figure 2:**
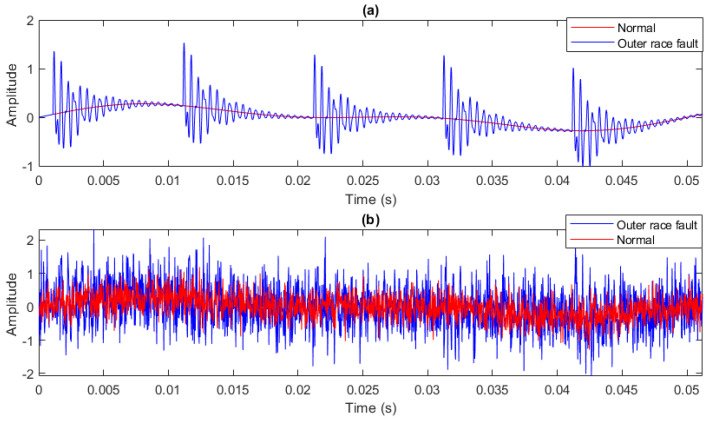
Simulated signals corresponding to the healthy and faulty bearings (**a**) without noise; (**b**) with noise.

Fifty independent healthy and faulty bearing signals with a length of 2048 data points and a sampling frequency of 40 kHz were simulated. Moreover, Gaussian noise was added to them with the variance ratio of signal to noise of 0.257 [52]. Figure 2 shows an example of these signals.

**Figure 3:**
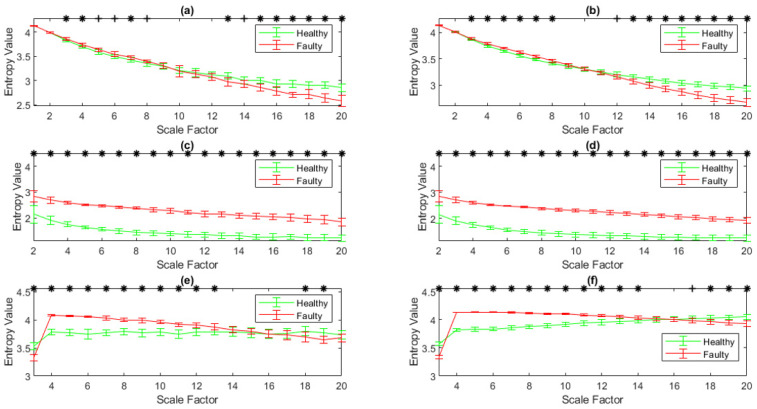
Comparison of 50 independent simulated signals corresponding to healthy and faulty bearings at 20 scales. (**a**) MDispEn; (**b**) RCMDispEn; (**c**) GMDispEn_2_; (**d**) RCGMDispEn_2_; (**e**) GMDispEn_3_; (**f**) RCGMDispEn_3_.

MDispEn, GMDispEn_2_, GMDispEn_3_, RCMDispEn, RCGMDispEn_2_, and RCGMDispEn_3,_ were calculated for the simulated signals, with the results displayed in Figure 3. In this figure, *p*-values smaller than 0.05 are identified with asterisks. According to Figure 3, RCMDispEn, RCGMDispEn_2_, and RCGMDispEn_3_ possess higher fault distinguishing capability than MDispEn, GMDispEn_2_, and GMDispEn_3_, respectively, and their results have a smaller standard deviation. Distinguishing abilities of the bearing faults using the generalized methods are also displayed.

Hedges’ *g* effect size [53] was used to evaluate the capability of these methods in discriminating the faulty from healthy ball bearing signals. The results are shown in Table 1. As can be seen, the GMDispEn_2_, GMDispEn_3_, RCGMDispEn_2_, and RCGMDispEn_3_ algorithms effectively show the differences between the healthy and the faulty conditions, similar to MDispEn and RCMDispEn. RCMDispEn, RCGMDispEn_2_, and RCGMDispEn_3_ have larger size effects and better fault separation capability than MDispEn, GMDispEn_2_, and GMDispEn_3_, respectively.

## 5. Analysis of the Experiments

### 5.1. Analysis of the Vibration Signals Acquired from the Case Western Reserve University Dataset

This section uses datasets from Case Western Reserve University (CWRU), US [54], with a sampling frequency of 48 kHz. The experimental set-up includes a three-phase induction motor, a torque transducer, and a dynamometer. The ball-bearing vibration signals were collected using an accelerometer installed on the motor housing at the drive end of the motor.

The signals consisted of 10 different fault conditions: healthy, ball fault, inner race fault, and outer race fault with intensities of 0.021″, 0.007″, and 0.014″. The shaft rotating speeds were 1772, 1750, and 1730 rpm.

A detailed description of the data set is shown in Table 2. For each condition, 180 samples with a length of 2048 were separated from the dataset signals with no overlap between any two samples. 

Specifically, 72, 18, and 90 signals were used for training, validation, and testing, respectively. MDispEn, GMDispEn_2_, GMDispEn_3_, RCMDispEn, RCGMDispEn_2_, and RCGMDispEn_3_ were calculated for all the signals, and their values were used in 20 scales as features for fault detection and classification. A binary vector was used as the target vector for every bearing condition. This binary vector had a length of 10 because 10 conditions were being studied. This research employed 10 FCM-ANFIS, each of which detected one element in the target vector.

**Figure 4:**
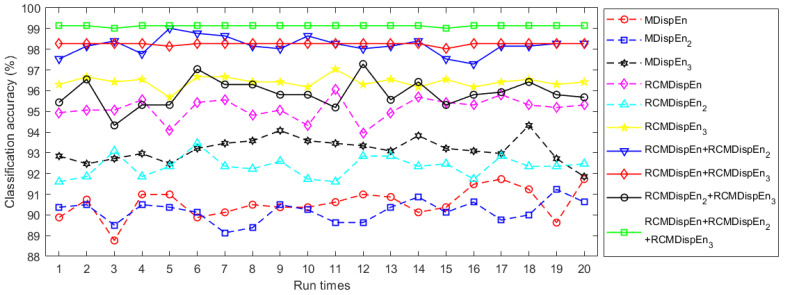
Classification accuracies of ball bearing fault diagnosis using ten different methods from the CWRU dataset.

The faulty conditions classification using multiclass FCM-ANFIS was performed 20 times with different inputs. The results of classifying these features are displayed in Figure 4 and Table 3. In this example, RCMDispEn, RCGMDispEn_2_, and RCGMDispEn_3_ performed better at classification than MDispEn, GMDispEn_2_, and GMDispEn_3_, respectively. Moreover, the simultaneous use of RCMDispEn, RCGMDispEn_2_, and RCGMDispEn_3_ as the classifier inputs produced the most accurate classification. Table 4 represents the confusion matrix of the best performance using these inputs.

### 5.2. Analysis of the Signals Acquired from the PHMAP 2021 Data Challenge Dataset

Part of the PHMAP 2021 data challenge dataset [55] was used in this section. The studied equipment consists of an oil injection screw compressor, containing a 15 kW and 3600 rpm motor and a 7200 rpm screw axis. This paper used data acquired using an accelerometer installed on the motor with a sampling frequency of 10,544 samples per second.

Three fault conditions were examined: (1) high Looseness of V-belt, (2) faulty bearing, and (3) fault-free condition. Three hundred independent signal samples with a length of 1024 samples were separated for each fault condition. 

**Figure 5:**
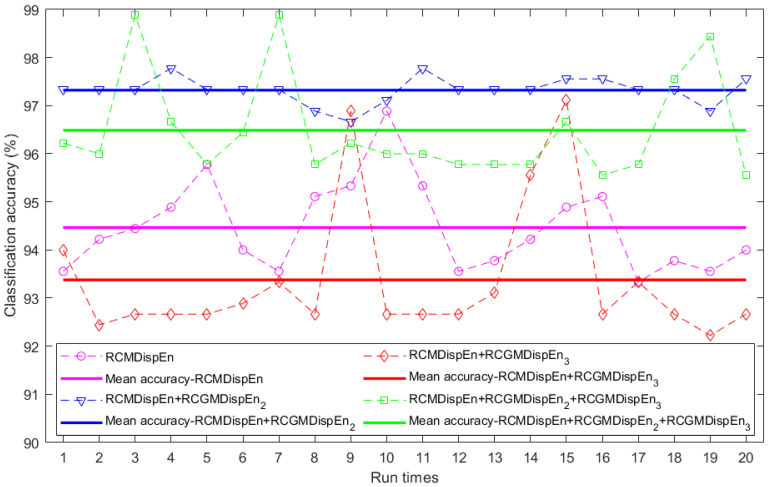
Results of classifying fault conditions: (1) high looseness of V-belt, (2) faulty bearing, and (3) fault-free condition using multiclass FCM-ANFIS with different inputs.

MDispEn, RCMDispEn, GMDispEn_2_, RCGMDispEn_2_, GMDispEn_3_, and RCGMDispEn_3_ were calculated for all the signals, and their values were used in 20 scales as features for fault detection and classification. For each condition, 120, 30, and 150 samples were used for training, validation, and testing, respectively. These data were classified 20 times using multiclass FCM-ANFIS. The results are displayed in Figure 5 and Table 5. As can be seen, the highest accuracy was achieved by the combined use of RCMDispEn, RCGMDispEn_2_, and RCGMDispEn_3_ as inputs. However, the mean accuracy of RCMDispEn and RCGMDispEn_2_ as simultaneous inputs was greater than that of other inputs. These results confirm the proposal of this paper regarding the use of generalized multiscale entropies with multiscale entropies to improve the results. The best classification results are displayed in Table 6.

### 5.3. Analysis of Vibration Signals Acquired from the Paderborn University Dataset

The data used in this section were from the ball bearing data collected in the Mechanical Engineering Construction and Drive Technology (KAt) Research data center, Paderborn University, Germany [56,57]. 

The classification of the datasets used in the present work is presented in Table 7, which represents three different fault conditions: (1) inner race damage, (2) outer race damage, and (3) healthy. The vibration signals corresponding to different bearing fault conditions under different operating conditions, shown in Table 8, were collected with a sampling frequency of 64,000 Hz.

A signal with a length of 1024 was separated from the beginning of every measured vibration signal, with 60 signals separated from each dataset, to obtain a total of 300 signals for each fault condition.

**Figure 6:**
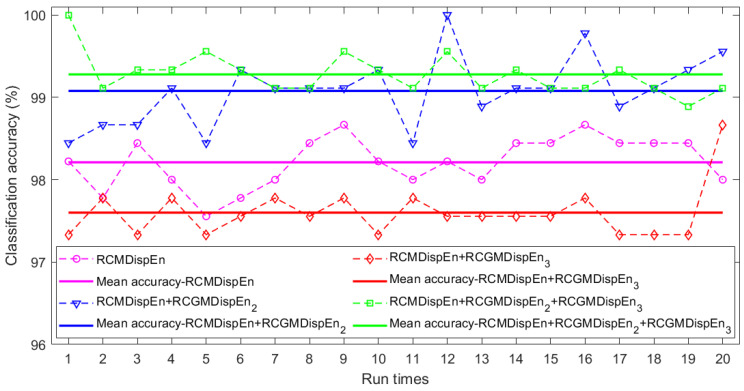
Results of classification of bearing fault signals using multiclass FCM-ANFIS with different inputs.

MDispEn, RCMDispEn, GMDispEn_2_, RCGMDispEn_2_, GMDispEn_3_, and RCGMDispEn_3_ were calculated for all the signals, and their values were used in 20 scales as features for fault detection and classification. For each condition, 120, 30, and 150 samples were used for training, validation, and testing, respectively. These data were classified 20 times using multiclass FCM-ANFIS. The results, displayed in Figure 6 and Table 9, confirm the suggestion made by the present study. Specifically, the highest classification accuracy corresponds to the features extracted by the combination of RCMDispEn, RCGMDispEn_2_, and RCGMDispEn_3_. Moreover, the smallest classification accuracy corresponds to the features extracted by RCMDispEn, RCGMDispEn_2_, and RCGMDispEn, separately.

## 6. Conclusions

The present paper investigated the simultaneous use of refined composite MDE based on different moments (i.e., first, second, and third moments, respectively, denote average, variance, and skewness respectively) to probe properties of the signals related to higher moments in bearing fault diagnosis. To this end, a bearing simulation example and three real datasets were utilized. Furthermore, the bearing fault classification was performed using multiclass FCM-ANFIS to examine the proposed technique. The results indicated that our developed RCGMDispEn_3_ and RCGMDispEn_2_ are more capable in separating bearing fault conditions compared to GMDispEn_3_ and GMDispEn_2_. Moreover, the combined use of RCGMDE, RCGMDE_2_, and RCGMDE_3_ produces better results than using one or two of these approaches in bearing fault diagnosis. The authors suggest investigating the potential of simultaneously using generalized multiscale and multiscale algorithms in other fields. 

## Figures and Tables

**Table 1:** Hedges’ g effect size of MDispEn, RCMDispEn, GMDispEn_2_, RCGMDispEn_2_, GMDispEn_3_, and RCGMDispEn_3_ in 20 scales on 50 independent healthy and faulty bearing signals.

Scale	Methods					
	MDispEn	RCMDispEn	GMDispEn_2_	RCGMDispEn_2_	GMDispEn_3_	RCGMDispEn_3_
1	0.6493	0.6493	-	-	-	-
2	0.2235	0.4915	2.2364	2.2569	-	-
3	1.1242	1.7345	5.0136	5.5878	3.2119	6.9364
4	1.1183	2.4674	10.8933	10.7749	7.9872	14.1039
5	1.0230	2.9302	16.6406	17.7200	6.6340	12.5567
6	1.0875	2.0787	15.7878	17.2561	4.6298	12.3608
7	0.9397	2.0842	11.0137	14.0667	5.9228	9.5684
8	1.0843	1.4531	1.8651	12.4676	4.3129	8.9234
9	0.2716	0.7559	10.4116	12.0355	3.8295	8.1357
10	0.0886	0.2386	9.0303	11.0007	3.4057	6.2796
11	0.3981	0.4373	8.7524	10.5785	2.7631	4.1878
12	0.6708	0.9714	7.4537	10.0266	2.0409	2.8769
13	1.4599	1.6187	7.3757	9.5024	1.8816	2.0764
14	0.9879	2.0674	6.8795	8.7588	0.5327	1.2610
15	1.9579	2.7560	6.7228	8.8263	0.3114	0.3482
16	1.7376	3.1014	6.3236	8.5332	0.0566	0.2229
17	3.2926	3.3136	6.1438	7.9683	0.3684	0.9012
18	1.9379	3.6472	4.9962	7.4363	1.1923	1.5027
19	3.2548	3.7864	4.4918	6.8664	1.8531	2.0079
20	2.7194	4.0228	4.2644	6.0740	0.7243	2.4493

**Table 2:** Description of bearing data set.

Bearing Condition	Defect Size (mm)	Label of Classification
Normal	0	1
Rolling element Fault	0.1778	2
Rolling element Fault	0.3556	3
Rolling element Fault	0.5334	4
Inner race Fault	0.1778	5
Inner race Fault	0.3556	6
Inner race Fault	0.5334	7
Outer race Fault	0.1778	8
Outer race Fault	0.5334	9

**Table 3:** The classification results of ball bearing faults using ten different inputs from the CWRU dataset.

	Accuracy (%)		
Features	Min	Mean	Max
MDispEn	88.7654	90.5679	91.7284
GMDispEn_2_	89.1358	90.1728	91.2346
GMDispEn_3_	91.8519	93.1605	94.3210
RCMDispEn	93.9506	95.1420	96.0494
RCGMDispEn_2_	91.6049	92.3457	93.4568
RCGMDispEn_3_	95.6790	96.4198	97.0370
RCMDispEn+ RCGMDispEn_2_	97.2840	98.1790	99.0123
RCMDispEn+ RCGMDispEn_3_	98.0247	98.2531	98.2716
RCGMDispEn_2_+ RCGMDispEn_3_	94.3210	95.8765	97.2840
RCMDispEn+RCGMDispEn_2_+ RCGMDispEn_3_	99.0123	99.1235	99.1358

**Table 4:** Confusion matrix of the testing set of the multiclass FCM-ANFIS using RCMDispEn, RCMDispEn, and RCMDispEn as the input.

		True Label									
		1	2	3	4	5	6	7	8	9	Sensitivity
**Predicted Label**	1	90	0	0	0	0	0	0	0	0	100
	2	0	90	0	0	0	0	0	0	0	100
	3	0	0	88	1	0	0	0	0	0	98.87
	4	0	0	0	87	0	2	0	0	0	97.75
	5	0	0	0	0	90	0	0	0	0	100
	6	0	0	0	1	0	88	0	0	0	98.88
	7	0	0	0	1	0	0	90	0	0	98.90
	8	0	0	0	0	0	0	0	90	0	100
	9	0	0	2	0	0	0	0	0	90	97.83
	**Precision**	100	100	97.78	96.67	100	97.78	100	100	100	AC * = 99.13

* AC is the accuracy.

**Table 5:** Results of classifying fault conditions: (1) high looseness of V-belt, (2) faulty bearing, and (3) fault-free condition using multiclass FCM-ANFIS with different inputs.

	Accuracy (%)		
Features	Min	Mean	Max
RCMDispEn	93.3333	94.4667	96.8889
RCMDispEn + RCGMDispEn_2_	96.6667	97.3222	97.7778
RCMDispEn + RCGMDispEn_3_	92.2222	93.3778	97.1111
RCMDispEn + RCGMDispEn_2_ + RCGMDispEn_3_	95.5556	96.4889	98.8889

**Table 6:** Most accurate classification of three fault conditions: (1) high looseness of V-belt, (2) faulty bearing, and (3) fault-free condition using RCMDispEn, RCMDispEn_2_, and RCMDispEn_3_ as inputs.

		True Condition			
		Belt Looseness High	Bearing Fault	Normal	Sensitivity (%)
Predicted condition	Belt Looseness High	148	0	0	100
	Bearing fault	0	150	3	98.04
	Normal	2	0	147	98.66
	Precision (%)	98.67	100	98	AC * = 98.89

* AC is the accuracy.

**Table 7:** Operating conditions.

No.	Rotational Speed [rpm]	Load Torque [Nm]	Radial Force [N]
1	1500	0.7	1000
2	1500	0.1	1000
3	1500	0.7	400

**Table 8:** Datasets used for three different bearing fault conditions.

	Type of Bearing		
	Healthy	Outer Ring Damage	Inner Ring Damage
Bearing Code	KI04	KA04	K001
	KI14	KA15	K002
	KI16	KA16	K003
	KI18	KA22	K004
	KI21	KA30	K005

**Table 9:** Classification results of bearing fault conditions: (1) inner race damage, (2) outer race damage, and (3) healthy.

	Accuracy (%)		
Features	Min	Mean	Max
RCMDispEn	97.5556	98.21111	98.6667
RCGMDispEn_2_	90.6667	91.34445	91.7778
RCGMDispEn_3_	86.6667	89.62222	92.2222
RCMDispEn + RCGMDispEn_2_	98.4444	99.07778	100
RCMDispEn + RCGMDispEn_3_	97.3333	97.6000	98.6667
RCMDispEn + RCGMDispEn_2_ + RCGMDispEn_3_	98.8889	99.27778	100

## Data Availability

The data that use in this study are openly available in CWRU datasets at https://engineering.case.edu/bearingdatacenter , PHMAP 2021 datasets at http://phmap.org/data-challenge , and KAt datasets at https://mb.uni-paderborn.de/kat/forschung/datacenter/bearing-datacenter/.
